# Novel Orthoreovirus from Mink, China, 2011

**DOI:** 10.3201/eid1912.130043

**Published:** 2013-12

**Authors:** Hai Lian, Ye Liu, Shoufeng Zhang, Fei Zhang, Rongliang Hu

**Affiliations:** Academy of Military Medical Sciences, Changchun, China

**Keywords:** Orthoreovirus, mink, diarrhea, reassortment, reovirus, swine, human, China

## Abstract

We identified a novel mink orthoreovirus, MRV1HB-A, which seems to be closely related to human strain MRV2tou05, which was isolated from 2 children with acute necrotizing encephalopathy in 2005. Evolution of this virus should be closely monitored so that prevention and control measures can be taken should it become more virulent.

The family *Reoviridae* is a diverse group of viruses with double-stranded RNA genomes contained within icosahedral, nonenveloped, double-layered, protein capsids (*1*). Members of the genus *Orthoreovirus* contain 10 genome segments and have been isolated from a wide variety of reptiles, birds, and mammals (including humans) (*1*,*2*). The mammalian orthoreoviruses (MRVs) have 4 major serotypes: type 1 Lang, type 2 Jones,, type 3 Dearing, and type 4 Ndelle (*3*,*4*), which can be differentiated by neutralization and hemagglutination inhibition assays (*5*,*6*). It is well known that reovirus genomes are prone to various types of genome alterations, including intragenic rearrangement and reassortment under laboratory and natural conditions (*7*,*8*). Reassortment events, involving exchange of genome segments between 2 viruses, which could lead to increased virulence, are major driving forces for reovirus genome molecular diversity and evolution (*9*,*10*). In 1975, natural reovirus infection in mink was first described in Germany (*11*). In 1992, Liu et al. also reported the isolation of a reovirus from the feces of mink with diarrhea in China (*12*). However, to our knowledge, no genetic evidence of MRV strains isolated from mink has been reported. 

We isolated a novel MRV strain (named MRV1HB-A) from a mink with diarrhea in Hebei Province in northern China. To track virus evolution and look for evidence of genetic reassortment, we used PCR sequencing and phylogenetic analysis to compare genetic relatedness of MRV1HB-A and other orthoreoviruses.

## The Study

**Figure 1:**
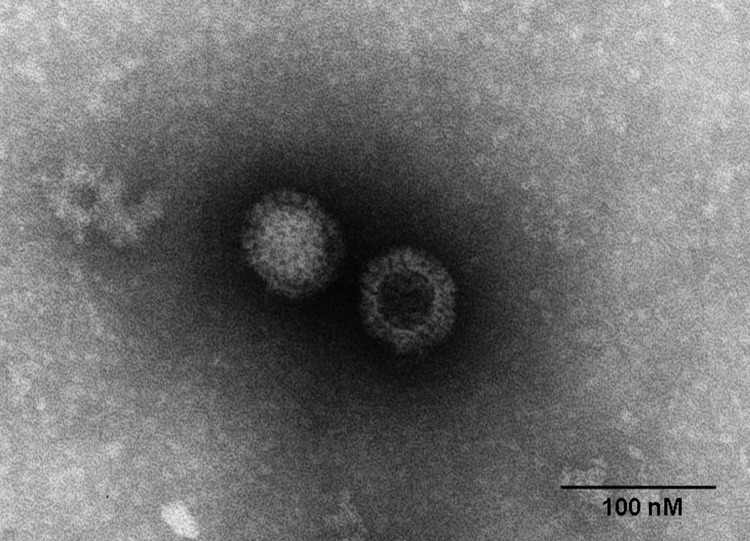
Electron micrograph of orthoreovirus MRV1-HB-A.

In 2011, minks on a breeding farm in Raoyang County, in southeastern Hebei Province, became ill with an unidentified disease. The illness rate was almost 100% among farmed minks (*Mustela vison*), although the death rate was <5%, mainly in minks <3 months of age. Clinical signs included anorexia, emaciation, unkempt fur, and diarrhea. PCR excluded all classical endemic and emerging viruses, mink enteritis virus, canine distemper virus, Aleutian mink disease virus, and mink coronavirus as the causative agent. To identify the cause of the disease, we homogenized fecal samples from affected minks in phosphate-buffered saline and subsequently inoculated the homogenate into FK81, Vero, and BHK-21 cells. On day 7, a strong cytopathic effect was observed in FK81 cells, including rounded and detached cells; on day 8, a similar cytopathic effect was observed in Vero cells; and on day 10, the cytopathic effect was observed in BHK-21 cells. Electron microscopy of infected cells demonstrated icosahedral, nonenveloped, viral particles characteristic of MRVs (Figure 1). The mink reovirus was able to hemagglutinate type O human erythrocytes (1% vol/vol) but not chicken, mouse, goose, or rabbit erythrocytes (1% vol/vol), a finding characteristic of MRVs. Using MRV-specific reverse transcription PCR assays, we obtained products of the predicted size of 416 bp for the polymerase large (L)1 gene regions. After direct sequencing of the PCR products, a BLAST (www.ncbi.nlm.nih.gov/blast/Blast.cgi) search showed the sequences to be authentic reovirus sequences, with closest similarity to those of the recently identified human MRV2tou05 strain, which had been isolated from 2 children with acute necrotizing encephalopathy in 2005 (*10*). These initial findings provide the genetic evidence that an enteric reovirus is shed in the diarrheal feces of mink, confirming a previous report suggesting MRV as an etiologic agent of acute viral enteritis in mink (*12*). We tested the pathogenicity of MRV1-HB-A by orally infecting 3-month-old minks at a dose of 3 × 10^5^ 50% tissue culture infective dose. Mucoid diarrhea was seen on day 5 after infection. The clinical signs were similar to those of naturally infected mink.

**Figure 2:**
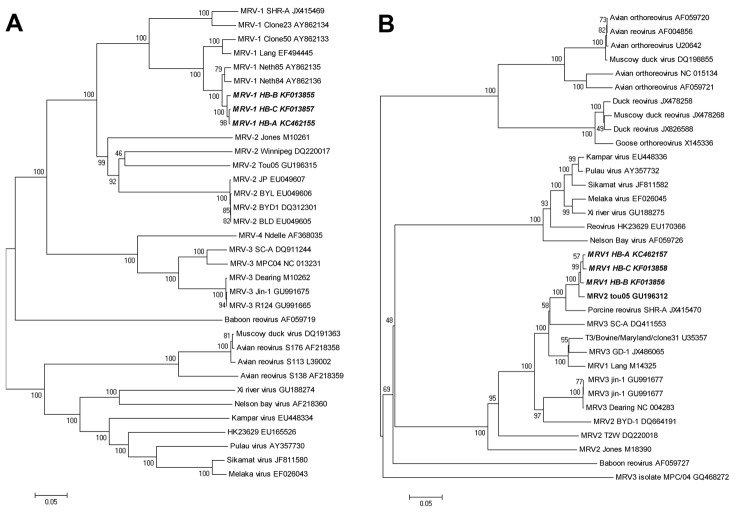
Phylogenetic tree and network based on the nucleotide sequences of the small (S)1 and S3 segments of orthoreoviruses. A and B) Neighbor-joining tree of multiple orthoreovirus species. Numbers at nodes indicate bootstrap values based on 1,000 replicates. Scale bar indicates nucleotide substitutions per site. A) S1 segment; B) S3 segment. C and D) Bayesian phylogenetic tree of multiple orthoreovirus species. Numbers close to branches correspond to posterior probabilities. Branch lengths are proportional to the number of nucleotide substitutions. Scale bar indicates substitutions per aligned position. C) S1 segment; D) S3 segment. D and F) Phylogenetic network of multiple orthoreovirus species. The Neighbor-Net graph was computed by using the SplitsTree 4.13.1 software (http://ab.inf.uni-tuebingen.de/data/software/splitstree4/download/welcome.html). E) S1 segment ; F) S3 segment. MRV, mammalian orthoreovirus. Strains sequenced in this study are indicated in ***boldface italics***.

To further identify the virus and its phylogeny, we amplified and sequenced the MRV1-HB-A L1–L3, medium (M)1–M3, and small (S)1–S4 genes (GenBank accession nos. KC462149– KC462158). Primers used for all 10 segments are shown in the Technical Appendix. The nucleotide sequences obtained for each segment were compared with those of other orthoreoviruses by using the ClustalX1.83 (www.clustal.org) programs. Phylogenetic relationship was assessed by using 3 approaches: Bayesian phylogenetic trees, neighbor-joining, and split network. Topology was essentially the same for Bayesian trees, neighbor-joining, and split network (Figure 2). Sequence analysis showed that the 6 segments (L1–L3, M2, M3, and S3) of MRV1-HB-A were closely related to those of the human MRV2tou05 strain and that the S1 and S2 segments were similar to those of other serotype 1 reoviruses that infect humans. The other 2 segments (M1 and S4) were closest to those of the SC-A strain, which was isolated from swine in 2006 in Sichuan, China (Table 1). The S1 segment of MRV1-HB-A is genetically similar to that of the human reovirus strains T1Neth/84 and T1Neth/85 (97% identity) isolated in the Netherlands in 1984 and 1985 (Figure 2). Nucleotide sequences of the prototype type 1 Lang and the MRV1-HB-A S1 genes shared 92% of positional identity, which provided sequence confirmation that this new isolate was a type 1 strain (Table 1). Otherwise, the S3 gene showed high identity with type 2 human reovirus Tou05 (98% identity) and porcine reovirus SHR-A (94% identity) strains (Figure 2). On the basis of these data, we conclude that the novel type 1 mink reovirus, designated MRV1-HB-A, might have originated from reassortment between human and swine strains.

**Table 1:** Nucleotide identities for segments of novel mink orthoreovirus MRV1HB-A, China*

MRV1HB-A RNA segment	Reovirus prototype strain, %					Human reovirus strain MRV2tou05, %		Swine reovirus strain, %	
	T1L	T2J	T3D	T4N		Tou05		SC-A	SHR-A
L1	89.5	75.8	89.5	90.2		**97.4**		96.9	91.3
L2	86.5	73	77.2	NA		**97.7**		95.9	84.5
L3	84.3	77.5	84.5	NA		**97.2**		96	85.6
M1	94.9	70.2	94.4	NA		90		**96.2**	94.9
M2	84.7	76.6	89.7	89.1		**96.4**		95.5	90.1
M3	85.7	71.2	85.1	NA		**97.3**		96.6	85.5
S1	**91.9**	58	40.6	42		58.6		40.9	73.3
S2	**96.2**	77.2	85.4	85.6		84.7		85.9	92.7
S3	90.7	74.4	85.5	NA		**97.8**		90	93.9
S4	87.9	79.9	87.4	91.2		94.9		**98.3**	78.1

*T1L, type1 Lang; T2J, type 2 Jones; T3D, type3 Dearing; T4N, type 4 Ndelle; L, large segment; NA, not available; M, medium segment; S, small segment. **Boldface** indicates high sequence identity.

Furthermore, we assessed the seroprevalence of mink reovirus antibodies in the human population and in 2 animal populations (mink and swine). Human serum samples were collected in Raoyao County People’s Hospital (n = 181) of Hebei Province and Chaoyang District People's Hospital (n = 128) of Jilin Province. The human serum was donated by students, faculty members, and university workers, when they underwent annual health checkups at the 2 hospitals. From swine, 122 serum samples were collected in Hebei (n = 66) and Guangdong (n = 56) Provinces. From mink, 78 serum samples were collected in Hebei (n = 48) and Jilin (n = 30) Provinces. All samples were tested by hemagglutination inhibition and microneutralization assay as described (*5*,*6*) by using the MRV1-HB-A isolated strains as antigen. Samples with titers ≥16 were considered seropositive after 2 independent assays; lower titers were considered nonspecific reactions. For all groups, hemagglutination inhibition and microneutralization assays demonstrated similar trends, although the percentage of samples that were positive for MRV1-HB-A antibodies by microneutralization was slightly lower than by hemagglutination inhibition assay (Table 2). Because these antibodies were found in some, but not all, serum samples tested from each species and because the seropositive rate differed among regions, these antibodies probably reflect actual infection with reovirus MRV1-HB-A strain or with other strains that include a similar S1 gene.

**Table 2:** Responses of human and animal serum samples to novel mink orthoreovirus MRV1-HB-A, China

Serum source, province	Hemagglutination inhibition, no. positive/no. tested (%)	Microneutralization, no. positive/no. tested (%)
Human		
Hebei	178/181 (98.3)	161/172 (93.6)
Jilin	116/128 (90.6)	97/117 (82.9)
Swine		
Hebei	33/66 (50)	27/59 (45.8)
Guangdong	50/56 (89.3)	44/52 (84.6)
Mink		
Hebei	48/48 (100)	46/46 (100)
Jilin	17/30 (56.7)	15/28 (53.6)

## Conclusions

Our study provides genomic evidence and molecular confirmation of a novel reovirus in mink. Although there is no direct evidence to prove the origin of MRV1-HB-A, the close genetic relationship of MRV1-HB-A with strains from humans and swine indicated a high probability that MRV1-HB-A resulted from a reassortment of human and swine strains. In view of the lack of sequence data for the reovirus from mink in public databases, addition of the complete genome sequencing information for the mink reovirus will aid in the characterization of mammalian reovirus diversity and evolution. Although MRVs were assumed to cause rather mild respiratory or gastrointestinal diseases, recent findings indicate the occurrence of higher virulent MRV strains in man and other mammals. To be prepared for the potential emergence of more virulent variants, we should carefully monitor virus evolution in real time. 

Technical Appendix Primers used for amplification of the genome of mink reovirus HB-A strain from China, 2011.
