# Supplementary material for: Novel Orthoreovirus from Mink, China, 2011
Source: Emerg Infect Dis. 2013 Dec;19(12):1985–8. doi: 10.3201/eid1912.130043 (PMC3840883; doi:10.3201/eid1912.130043)
Supplement: Technical Appendix — Primers used for amplification of the genome of mink reovirus HB-A strain from China, 2011. [file 13-0043-Techapp-s1.pdf]

Article DOI: http://dx.doi.org/10.3201/eid1912.130043

Novel Orthoreovirus from Mink, China, 2011

Technical Appendix

Technical Appendix Table. Primers used for amplification of the genome of mink reovirus HB-A strain from China, 2011*

Gene, primer name	Primer sequence, 5′→3′
L1 forward	GCTACACGTTCCACGACAAT
L1 reverse	TGAGTTGACGCACCACGACCCA
L2 forward	ATGGCGAACGTTTGGGGAGT
L2 reverse	GATGAATTAGGCACGCTCACG
L3 forward	TAATCGTCAGGATGAAGCGGA
L3 reverse	TGAATCGGCCCAACTAGCAT
M1 forward	ATGGCTTACATCGCAGTTCCT
M! reverse	CGTAGTCTTAGCCCGCCCC
M2 forward	TAATCTGCTGACCGTCACTC
M2 reverse	GTGCCTGCATCCCTTAACC
M3 forward	CGTGGTCATGGCTTCATTC
M3 reverse	GATGAATAGGGGTCGGGAA
S2 forward	GCTATTCGCTGGTCAGTTATGGC
S2 reverse	GATGAATGTGTGGTCAGTCGTGAG
S3 forward	GCTAAAGTCACACCTGTCGTCGTC
S3 reverse	GATGATTAGGCGCCACCCACCAC
S4 forward	GCTATTTTTGCCTCTTCCCAAACGTTGTCG
S4 reverse	GATGAATGGAGCCTGTCCCACGTCACACC
S1 forward	GCTATTCGCGCCTATGGA
S1 reverse	GATGAAATGCCCCAGTGC

*L, large; M, medium; S, small.
